# Differential Effects of Oleic and Palmitic Acids on Lipid Droplet-Mitochondria Interaction in the Hepatic Cell Line HepG2

**DOI:** 10.3389/fnut.2021.775382

**Published:** 2021-11-12

**Authors:** Andrea Eynaudi, Francisco Díaz-Castro, Juan Carlos Bórquez, Roberto Bravo-Sagua, Valentina Parra, Rodrigo Troncoso

**Affiliations:** ^1^Laboratorio de Investigación en Nutrición y Actividad Física (LABINAF), Instituto de Nutrición y Tecnología de los Alimentos (INTA), Universidad de Chile, Santiago, Chile; ^2^Laboratorio de Obesidad y Metabolismo Energético (OMEGA), Instituto de Nutrición y Tecnología de los Alimentos (INTA), Universidad de Chile, Santiago, Chile; ^3^Advanced Center for Chronic Diseases (ACCDiS), Facultad de Ciencias Químicas y Farmacéuticas, Universidad de Chile, Santiago, Chile; ^4^Red de Investigación en Envejecimiento Saludable, Consorcio de Universidades del Estado de Chile, Santiago, Chile; ^5^Red Para el Estudio de Enfermedades Cardiopulmonares de Alta Letalidad (REECPAL), Universidad de Chile, Santiago, Chile

**Keywords:** lipid droplets, mitochondria, fatty acids, hepatocytes, oxygen consumption

## Abstract

Fatty acid overload, either of the saturated palmitic acid (PA) or the unsaturated oleic acid (OA), causes triglyceride accumulation into specialized organelles termed lipid droplets (LD). However, only PA overload leads to liver damage mediated by mitochondrial dysfunction. Whether these divergent outcomes stem from differential effects of PA and OA on LD and mitochondria joint dynamics remains to be uncovered. Here, we contrast how both fatty acids impact the morphology and interaction between both organelles and mitochondrial bioenergetics in HepG2 cells. Using confocal microscopy, we showed that short-term (2–24 h) OA overload promotes more and bigger LD accumulation than PA. Oxygen polarography indicated that both treatments stimulated mitochondrial respiration; however, OA favored an overall build-up of the mitochondrial potential, and PA evoked mitochondrial fragmentation, concomitant with an ATP-oriented metabolism. Even though PA-induced a lesser increase in LD-mitochondria proximity than OA, those LD associated with highly active mitochondria suggest that they interact mainly to fuel fatty acid oxidation and ATP synthesis (that is, metabolically “active” LD). On the contrary, OA overload seemingly stimulated LD-mitochondria interaction mainly for LD growth (thus metabolically “passive” LDs). In sum, these differences point out that OA readily accumulates in LD, likely reducing their toxicity, while PA preferably stimulates mitochondrial oxidative metabolism, which may contribute to liver damage progression.

## Introduction

Non-alcoholic fatty liver disease (NAFLD) is the most common liver disease, affecting 15–30% of the world population. It has a direct association with obesity, insulin resistance, and cardiovascular diseases (1) and characterizes by excessive fat accumulation in the liver (>5% in hepatocytes) (2). Therefore, its development tightly relates to fatty acid (FA) intake. On this regard, evidence supports the idea that saturated FA predisposes to hepatic lipid accumulation (termed steatosis), while unsaturated FA could be protective (3).

The most abundant saturated and monounsaturated FA in the Western diet are palmitic (PA, C16:0) and oleic acid (OA, C18:1 n-9) (4). During intestinal absorption, they are esterified into triglycerides (TG), and then delivered to the liver, which subsequently distributes them to other organs. However, excessive levels of either PA and OA lead to steatosis but with distinct cellular outcomes (5, 6). On the one hand, PA treatment causes liver lipotoxicity via oxidative stress, resulting in endoplasmic reticulum (ER) and mitochondrial dysfunction, and ultimately cell demise (5–7). In contrast, primary cultures of mouse hepatocytes treated with OA do not display either increased generation of oxygen radicals or signs of mitochondrial dysfunction or apoptosis. Moreover, in the hepatocyte-derived cell line HepG2, OA even prevented PA-induced liver lipotoxicity (6, 7).

Acting as protection against lipotoxicity, lipid droplets (LD) serve as TG deposits, thereby preventing fat accumulation in other cell compartments (8). Structurally, they comprise a nucleus of neutral lipids, mostly TG, surrounded by a monolayer of phospholipids and specific coating proteins, such as those belonging to the perilipin (PLIN) family. LD are highly dynamic organelles, varying in number and size according to storage requirements. Conversely, LD also hydrolyze TG, thus releasing free fatty acids, which serve as energy sources through their degradation (9). Fatty acid degradation takes place at mitochondria, which produce ATP through oxygen-driven oxidation (10). Like LDs, mitochondria vary in number and size to cope with varying nutritional scenarios, such as fasting and physical activity (11). In this sense, studies suggest that smaller mitochondria are more oxidative and thus, synthesize ATP more efficiently (8).

**Figure 1:**
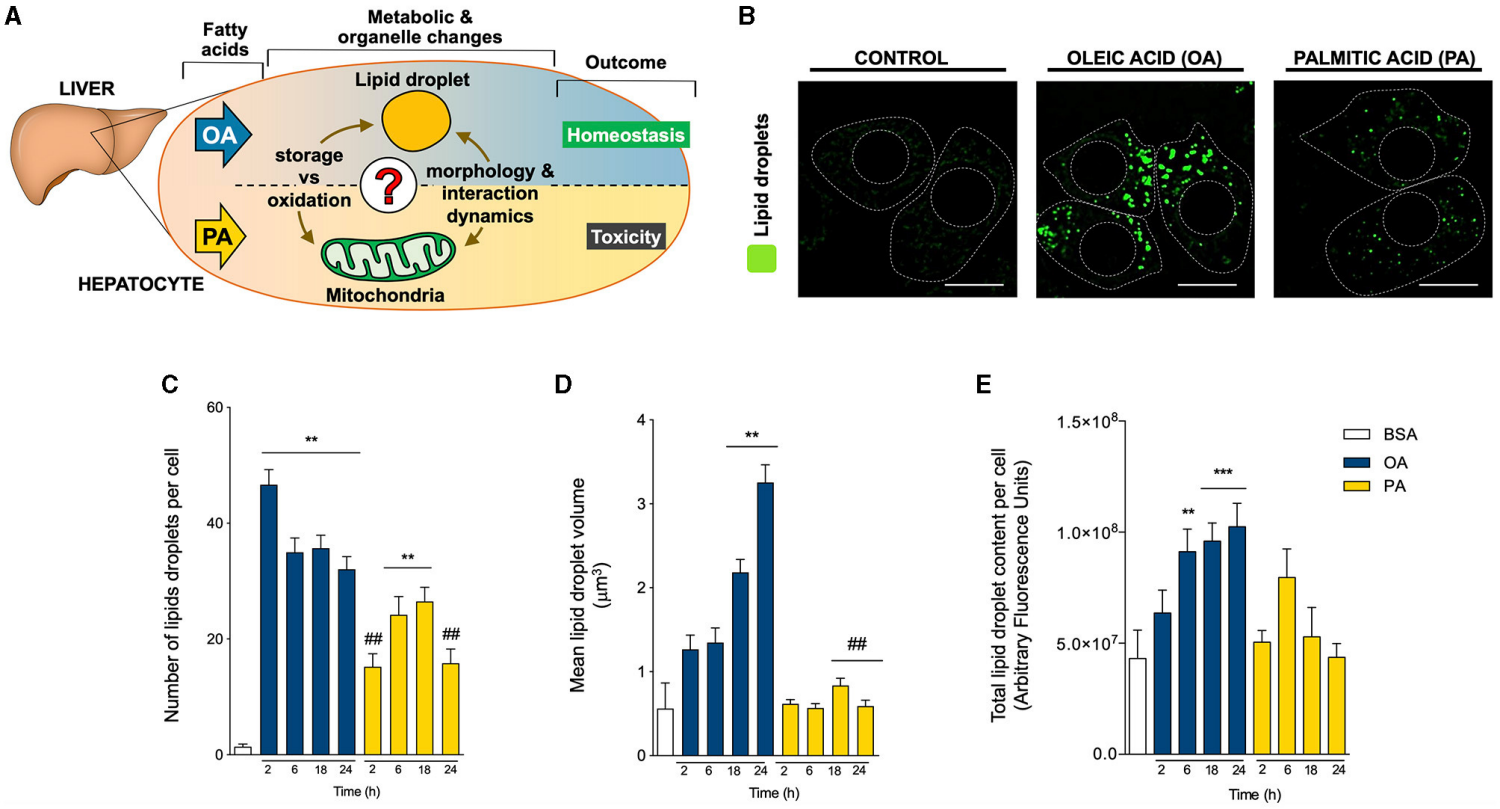
Oleic and palmitic acids differentially remodel lipid droplet morphology in HepG2 cells. **(A)** In the liver, oleic acid (OA) overload seemingly does not damage hepatocyte function, while palmitic acid (PA) is evidently toxic. To date, the underlying mechanism of this difference remains unknown in terms of fatty acid metabolism and mitochondria-lipid droplet interactions and morphology. **(B)** Representative confocal imaging of HepG2 cells treated with 200 μM of OA or PA during 18 h and stained with Bodipy 493/503 (green) for lipid droplet (LD) visualization. Segmented lines represent the cellular and nuclear contours. Scale bar: 10 μm. **(C)** Quantification of the number of LDs per cell in images treated as in **A** for 0–24 h. **(D)** Mean individual volume of LDs in HepG2 cells treated as in **A** for 0–24 h. **(E)** Quantification of the total Bodipy 493/503 fluorescence in HepG2 cells treated as in **A** for 0–24 h. Data are from *N* = 6–20 cells analyzed from three independent experiments. Results are shown as mean ± SEM; ^*^p < 0.05; ^**^p < 0.01; ^***^p < 0.001 vs. BSA; ^#^ *p* < 0.05; and ^##^ *p* < 0.01 vs. OA at the same time conditions.

Apart from individual dynamics, mounting evidence shows that mitochondria and LD physically interact, especially in tissues with a high capacity for fatty acid oxidation and storage, such as the liver, heart, brown adipose tissue, and skeletal muscle (8, 12). For instance, PLIN5 mediates LD-mitochondrial interaction in the mouse liver cell line AML12 (13) and cardiac tissue of mice, resulting in LD expansion and a decrease in fatty acid oxidation (14). Likewise, in adipose tissue, PLIN2 binds the mitochondrial protein MIGA2, thus bridging mitochondria and LD (15). Furthermore, PLIN5 and PLIN2 form a complex, which favors mitochondrial recruitment to the surface of LDs in cardiomyocytes (14). In adipose tissue, PLIN1 reportedly promotes LD interaction with mitochondria by binding to the proteins MFN2 and OPA1 at the mitochondrial surface (16, 17). Interestingly, Benador et al. attained similar results about LD-mitochondria association in brown adipose tissue (8). In hepatocytes, the physiological role of LD-mitochondria interaction is yet to be unveiled. Also, despite being the most abundant dietary fatty acids, little is known about the differences between the effect of PA and OA on LD morphology and contact with mitochondria in hepatocytes (Figure 1A). Therefore, the objective of this work was to contrast how PA and OA impact LD-mitochondria dynamics and mitochondrial bioenergetics and how these processes are associated with the development of hepatic steatosis.

## Methods

### Cell Culture and Treatments

The HepG2 cell line was obtained from the American Type Culture Collection (ATCC HB-8065). Cells were cultured in Dulbecco's modified Eagle's medium (DMEM 11965-09; Thermo Fisher Scientific), supplemented with 10% fetal bovine serum (FBS), 1% penicillin-streptomycin antibiotics (Biological Industries) and maintained at 37 °C with a 5% CO_2_ atmosphere. Hepatocytes were exposed to 200 μM OA (P1383, Sigma-Aldrich) or 200 μM PA (P0500; Sigma-Aldrich) conjugated to fatty acid-free bovine serum albumin (BSA) (A8806; Sigma-Aldrich) for 2, 6, 18 and 24 h, as previously described (18). Cells treated with BSA were used as controls.

### Western Blot Analysis

Cell protein extracts were obtained using T-PER lysis buffer (78510, Thermo Fisher Scientific), supplemented with protease (#04693159001, Roche) and phosphatase (#04906845001, Roche) inhibitors. Homogenates were centrifuged at 14,500 G for 10 min, and the supernatants were collected for measuring protein concentrations with the BCA protein assay (23227, Thermo Fisher Scientific). Protein extracts of 50 μg were resolved in 10% SDS-polyacrylamide gels, transferred onto PVDF membranes previously activated with methanol, and blotted overnight with primary antibodies at 4 °C. The antibodies and dilutions were: anti-PLIN2 (1:1000; #MA5-24797, Thermo Fisher Scientific), anti-PLIN5 (1:1000; #GP31, Progen), and anti-MFN2 (1:1000; #ab50838, Abcam). The membranes were then incubated for 1 h with the following secondary HRP-coupled antibodies: anti-mouse (1:5000; #402335, Calbiochem), anti-guinea pig (1:5000; #ab6908, Abcam) or anti-rabbit (1:5000; #401315, Calbiochem) and revealed with the EZ-ECL detection kit (DW1029, Biological Industries). A C-DiGit® Blot Scanner and the Image Studio software version 3.1 (LI-COR) were used for image acquisition and densitometric analysis. GAPDH (1:5000; #MAB374, Sigma-Aldrich) was used as a loading control.

### ATP Measurements

Intracellular ATP content was determined using the luciferin/luciferase-based ATP detection kit CellTiter-Glo Luminescent Cell Viability Assay (Promega) following the manufacturer's instructions as described in (19). Briefly, HepG2 cells were cultured in 96-well Petri dishes and washed 3 times with PBS before incubation with the reagent. Sample luminescence was quantified in a Synergy 2 microplate reader (BioTek Instruments). Data were normalized as fold of changes over control. Treatment with oligomycin (5 μg/mL) for 3 h was used as a negative control.

### Oxygen Consumption

Cells were seeded in 60 mm Petri dishes at 80% confluence and treated according to the experiment. After the different treatments, measurements were performed as previously described (20–22). In brief, cells were washed with PBS, trypsinized for 3 min, and centrifuged at 200 G for 5 min. Then, the cells were resuspended in PBS and placed in the chamber of a Clark electrode (Oxygraph Plus, Hansatech). Basal respiration, proton leak, or ATP-unlinked respiration (Oligomycin, 400 μM) and uncoupled respiration (FCCP 20 μM) were measured sequentially for 3 min. Data obtained were standardized to basal control respiration.

### Microscopy

Cells were plated on 12 mm Petri dishes and treated according to the experiment. To label LDs and mitochondria, cells were stained with BODIPY 493/503 (2 μM; D3922, Invitrogen) and MitoTracker Orange (400 nM; M7510, Invitrogen) for 25 min at 37 °C. Then, the cells were fixed with 4% paraformaldehyde and 0.01% Hoechst in PBS, and mounted in Dako Fluorescence Mounting Medium (S3023, Dako-Agilent). Fixed cells were imaged using a Nikon C2 Plus-SiR confocal microscope. 6–20 cells were registered for 3 independent experiments.

### Image Analysis

Images acquired were deconvoluted, background-subtracted, thresholded, and analyzed with ImageJ software (NIH). LDs and mitochondria number and individual volume were quantified using the 3D Object Counter plugin, as previously described (20–23). LD-mitochondria colocalization was determined within one focal plane using the JACoP plugin (20, 22, 23). The mitochondrial potential was defined in relation to MitoTracker Orange fluorescence intensity, and was analyzed within a single plane at the cell equator with the Analyze Particles function (20, 22). The mitochondrial potential of bound LD and non-bound-LD was performed by constructing a compartment consisting of 10 z-planes of the sites where mitochondria colocalize with LDs. Then, the intersected or excluded mitochondrial fluorescence of bound LD and non-bound-LD was quantified, respectively. Image intersections were obtained using the Image Calculator command of ImageJ (“AND” operator) (22).

### Statistical Analysis

All statistical analyses were performed using GraphPad Prism software, version 6 (San Diego, CA, USA). Data are expressed as mean ± SEM of at least three independent experiments. Data were analyzed by one-way ANOVA, and, when appropriate, comparisons between groups were performed using Tukey's or Dunnett's *post-hoc* tests. A two-tailed Pearson's coefficient was used for correlation analysis. Differences were considered significant at *P* < 0.05 (that is, a confidence level of 95%, α = 0.05).

## Results

### Oleic and Palmitic Acids Have Differential Effects on LD and Mitochondrial Dynamics

To evaluate whether OA or PA affects the morphology of LDs, HepG2 hepatocytes were treated with OA or PA between 0-24 h. Whereas OA (Figure 1B) stimulated the appearance of big and bright droplets in hepatocytes, mainly in the central region of the HepG2 cells, PA triggered the emergence of evenly distributed smaller droplets. To quantify this effect, LD morphology was also assessed according to the number and volume of individual isolated elements through 3D reconstitution of confocal stacks (20–23) and the total fluorescence of BODIPY. Compared to BSA, OA significantly increased the number of LDs per cell at all the times analyzed, reaching a ~30-fold increase as early as 2 h after treatment (Figure 1C). Subsequently, OA also led to a gradual increase in the volume of the LDs, reaching ~4 and ~6-fold, compared to controls after 18 and 24 h, respectively (Figure 1D). In terms of total BODIPY fluorescence, only OA triggered a significant increase in the fluorescence after 6 h of treatment; moreover, this increase was maintained at later times of 18 and 24 h (Figure 1E). These results agree with the rapid nucleation of new LDs, which later grow over time. On the other hand, treatment with PA led to a slower increase in the number of LD per cell, which was significant (~20-fold) at 6 and 18 h of treatment (Figure 1C). In contrast to OA, PA did not change the volume of the LDs, suggesting slow nucleation of new LD without a growing phase (Figure 1D).

**Figure 2:**
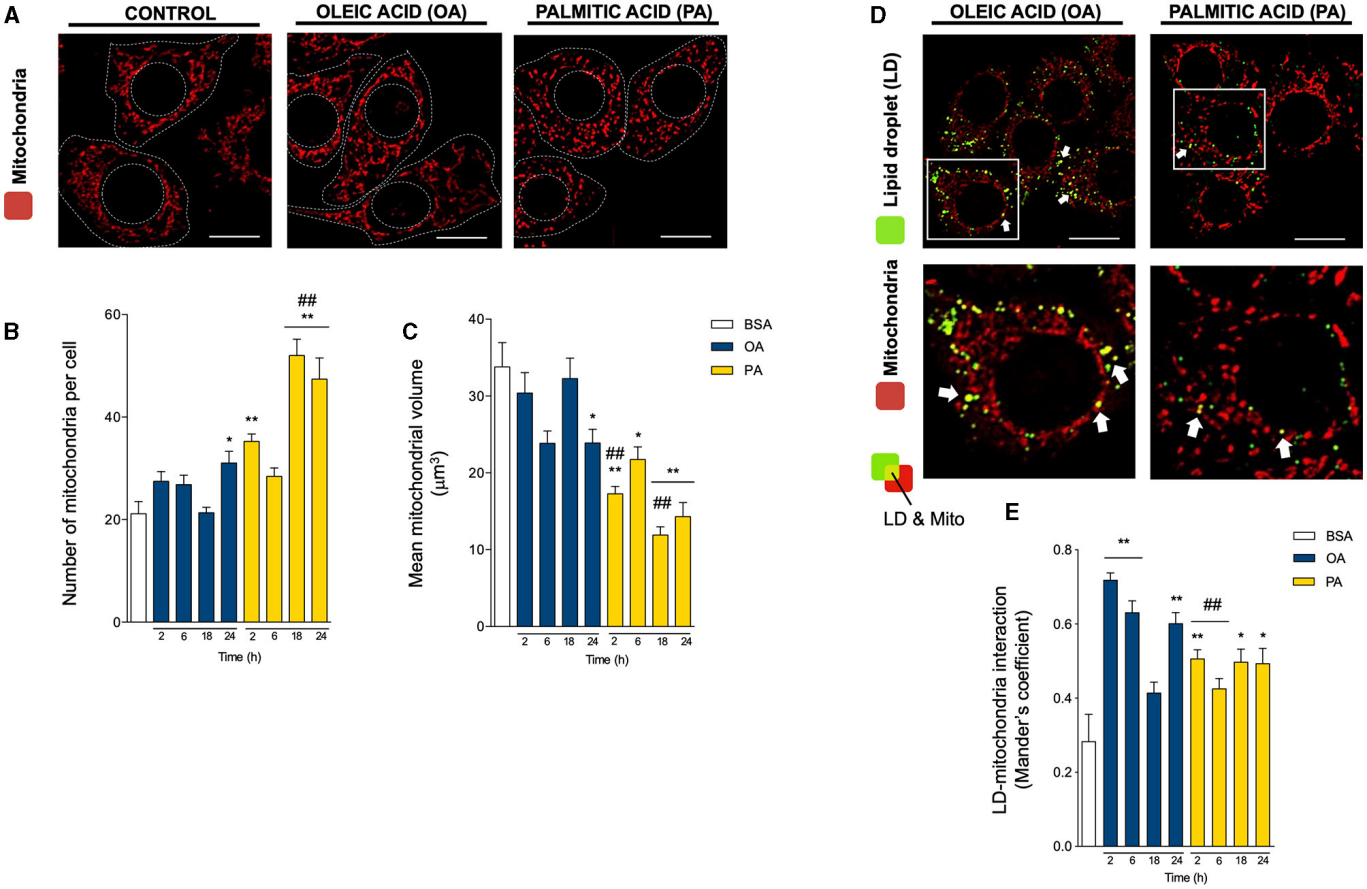
Oleic and palmitic acids differentially remodel mitochondrial morphology and their interaction with lipid droplets in HepG2 cells. **(A)** Representative confocal imaging of HepG2 cells treated with 200 μM of oleic acid (OA) or palmitic acid (PA) during 18 h and stained with MitoTracker Orange (red) for mitochondria visualization. Segmented lines represent the cellular and nuclear contours. Scale bar: 10 μm. **(B)** Quantification of the number of mitochondria per cell in images treated as in **A** for 0–24 h. **(C)** Mean individual mitochondrial volume of HepG2 cells treated as in **A** for 0–24 h. **(D)** Representative merged confocal imaging of HepG2 cells treated with 200 μM of OA or PA during 6 h and stained with Bodipy 493/503 (green) and MitoTracker Orange (red) for lipid droplet (LD) and mitochondria visualization, respectively. Colocalized areas are shown in yellow. White arrow marks spots of high colocalization. Scale bar: 10 μm. **(E)** Quantification of LD-mitochondria colocalization using Manders' coefficient for cell images obtained as described in **D**. Data are from *N* = 6–20 cells analyzed from three independent experiments. Scale bar: 10 μm. Results are shown as mean ± SEM; ^*^ *p* < 0.05; ^**^ *p* < 0.01 vs. BSA; and ^##^ *p* < 0.01 vs. OA at the same time conditions.

We next evaluated mitochondrial morphology in HepG2 hepatocytes using the mitochondrial-specific MitoTracker Orange probe (400 nM, 25 min) and 3D-reconstruction imaging (Figures 2A–C), as we have previously reported (21, 23). In contrast to the steep changes in LD morphology, OA only caused a slight but significant increase in the number of mitochondria per cell, concomitant with a decrease in their volume after 24 h, indicative of the induction of mitochondrial fission (Figures 2B,C). Meanwhile, PA treatment triggered a faster and more intense process of mitochondrial fragmentation, noticeable after 2 h, reaching a ~2-fold increase in the number of mitochondria per cell and a significant ~2-fold reduction in mitochondrial volume (Figures 2B,C). The magnitude of these differences reveals maintenance in the total size of the mitochondrial network, mainly suggesting changes in the mitochondrial fusion/fission equilibrium. Our results with PA are consistent with their reported effects on mitochondrial morphology in other cell types (24–26).

To evaluate whether the changes in organelle dynamics affect their physical coupling, we then assessed LD-mitochondria proximity, evaluated as Mander's colocalization coefficients between LD and mitochondria (20, 22, 27) (Figures 2D,E). Both fatty acids increased LD-mitochondria proximity starting at 2 h. However, OA treatment induced a significantly higher increase at 2 h (>2-fold), which later decreased to values similar to PA (~1.5-fold). Taken together, these results suggest that OA treatment triggers the early appearance of more and bigger LDs than PA, which are in closer contact with mitochondria in HepG2 hepatocytes.

### Oleic and Palmitic Acids Evoke Different Profiles of LD-Mitochondria Interaction Proteins

**Figure 3:**
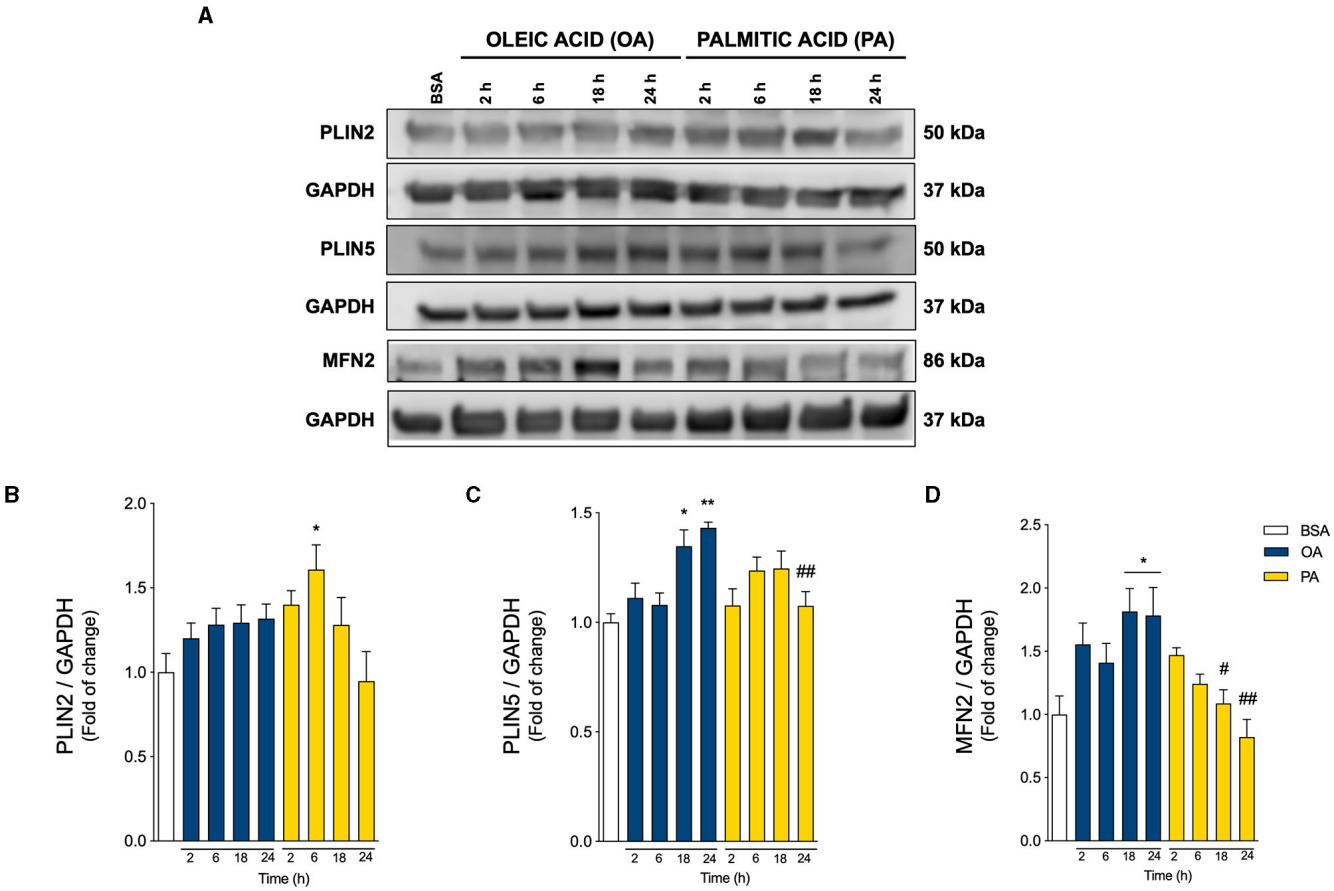
Oleic and palmitic acids induce different patterns of lipid droplet-mitochondria tether proteins in HepG2 cells. **(A)** Representative Western blots of the proteins associated with lipid droplet (LD)-mitochondria physical interaction: Perilipin 2 and 5 (PLIN2, PLIN5), and Mitofusin 2 (MFN2) in HepG2 cells treated with 200 μM of oleic acid (OA) or palmitic acid (PA) for 0–24 h. GAPDH was used as a loading control. **(B–D)** Densitometric quantification of the proteins indicated in **A**. Data are expressed as mean ± SEM of *N* = 7 for PLIN2 and *N* = 4 for PLIN5 and MFN2. ^*^ *p* < 0.05; ^**^ *p* < 0.01 vs. BSA; ^#^ *p* < 0.05; and ^##^ *p* < 0.01 vs. OA at the same time conditions.

PLINs are the main structural proteins of LDs, and determine their structure, metabolism, and interaction with other organelles (28, 29). While PLIN2 is ubiquitously expressed, PLIN5 is especially enriched in tissues with high levels of mitochondrial oxidation, such as the liver (29). PLIN5 reportedly regulates mitochondrial recruitment to LD, thereby regulating mitochondrial metabolism (8, 29, 30). On the other hand, MFN2 not only promotes mitochondrial fusion and boosts mitochondrial metabolism (31, 32), but also binds to PLIN1 to facilitate the LD-mitochondria interaction in adipose tissue (17, 33). Due to their importance, we measured their relative abundance in our experimental model through Western blot analysis (Figure 3A). As shown in Figures 3A,B, PA triggered a significant acute increase in PLIN2 at 6 h of treatment, without altering MFN2 or PLIN5, compared to control. On the other hand, OA increased both PLIN5 and MFN2 (Figures 3A,C,D) at later times (18–24 h). These results suggest that OA promotes PLIN5-MFN2-mediated LD-mitochondria interaction, while PA favors the presence of PLIN2 at the surface of LDs.

### Oleic and Palmitic Acids Differentially Modulate Mitochondrial Oxidative Function

**Figure 4:**
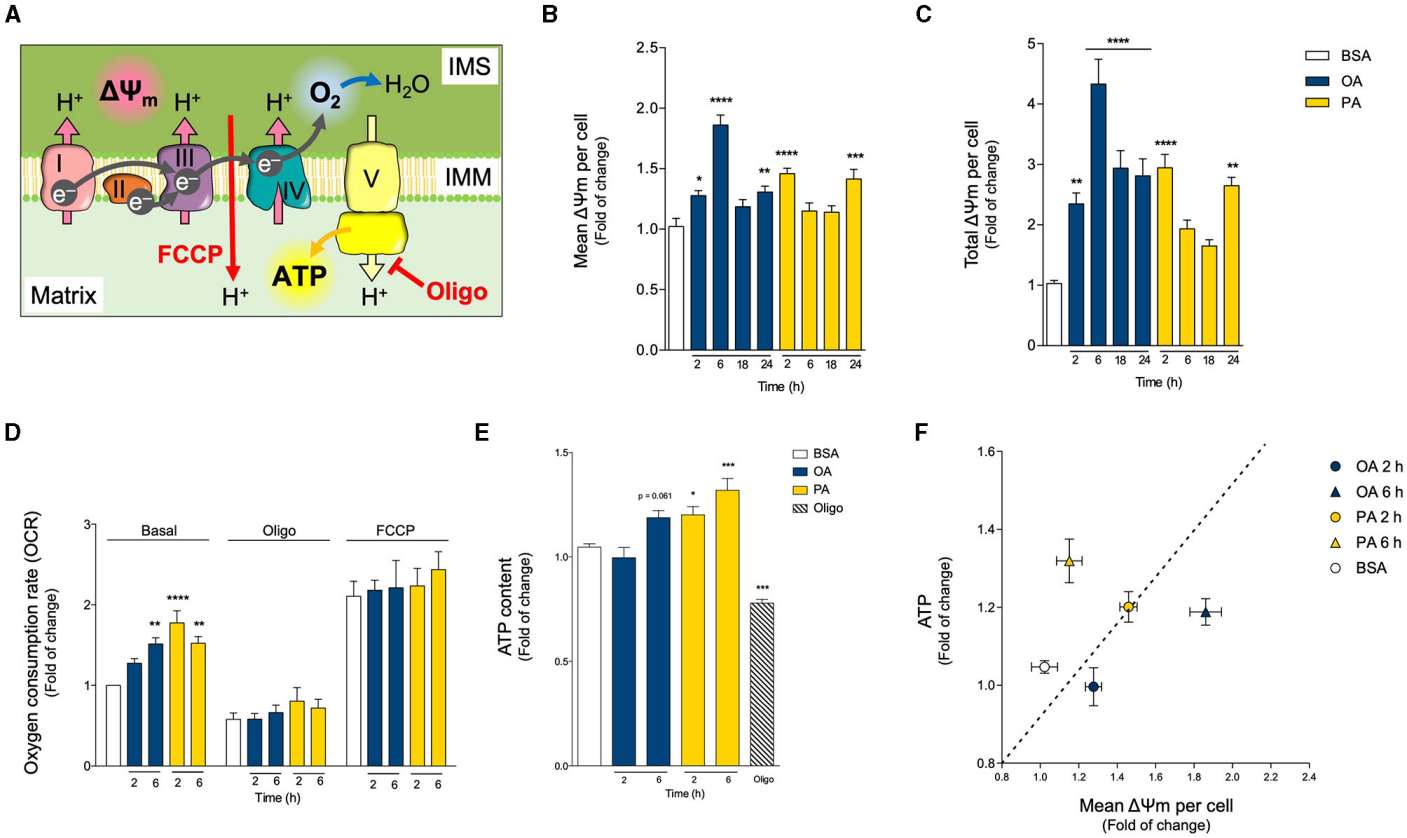
Oleic and palmitic acids differentially modulate mitochondrial bioenergetics in HepG2 cells. **(A)** Schematics of mitochondrial respiration through the mitochondrial matrix and the intermembrane space (IMS). Complexes I, III and IV pump H^+^ across the inner mitochondrial membrane (IMM), generating the mitochondrial transmembrane potential (ΔΨm), and ultimately, transforming O_2_ into H_2_O. Then, complex V synthesizes ATP using the proton gradient of the ΔΨm. Oligomycin (Oligo) inhibits Complex V, thus leading to the build-up of the ΔΨm, which hampers mitochondrial respiration. FCCP, on the other hand, dissipates the ΔΨm, thereby eliminating the electrochemical restraint on mitochondrial respiration. **(B,C)** Mean and whole-cell Δψm of HepG2 cells treated with 200 μM of oleic acid (OA) or palmitic acid (PA) for 0–24 h, measured using MitoTracker Orange and confocal fluorescence microscopy. Data are from *N* = 6–20 cells analyzed from three independent experiments. **(D)** Oxygen consumption rate (OCR) of cells treated as in A, measured after 2 and 6 h of treatment. Baseline respiration, non-ATP-associated respiration (Oligo 400 μM), and the maximal respiratory capacity (FCCP 20 μM) were measured sequentially for 3 min (*N* = 5). **(E)** Intracellular ATP levels of cells treated as in **A**, quantified after 2 and 6 h of treatment. Oligo 5 μg/mL for 3 h was used as a positive experimental control (*N* = 5). **(F)** Mitochondrial efficiency expressed as ATP levels vs. OCR at 2 h of OA or PA treatments. Results are shown as mean ± SEM; ^*^p < 0.05; ^**^p < 0.01; ^***^p < 0.001; and ^****^p < 0.0001 vs. BSA.

Mitochondria act as oxidative centers for different metabolites, such as fatty acids, which ultimately fuel a chain of redox reactions driven by oxygen-mediated oxidation. The protein complexes that catalyze this redox process (termed the electron transport chain, ETC) pump protons across the inner mitochondrial membrane. This creates an electrochemical gradient, which entails the generation of a mitochondrial transmembrane potential (Δψm). The resulting proton-motive force drives ATP production by transporting the protons back across the mitochondrial inner membrane through the ATP synthase enzyme (23, 34). Of note, this mechanism implies that ATP production dissipates the Δψm (Figure 4A). Thus, to characterize the differential effects of OA and PA treatments on mitochondrial metabolism, we analyzed three defining parameters of mitochondrial bioenergetics (34): Δψm, O_2_ consumption, and ATP levels.

We first evaluated the Δψm by using the mitochondria-specific MitoTracker Orange potentiometric probe and confocal microscopy. Because PA treatment triggers mitochondrial fragmentation and a concomitant decrease in mitochondrial volume, to quantify the Δψm, we evaluated both the mean mitochondrial fluorescence and the total fluorescence per cell of MitoTracker Orange. The first fluorescence parameter addresses the bioenergetic state of individual mitochondria, and the second parameter evaluates the metabolic state of the cell as a whole. Figures 4B,C shows that both OA and PA treatments increased the mean and total fluorescence levels of MitoTracker Orange, starting at 2 h, indicating a boost in the Δψm. In the case of OA, the Δψm peaked at 6 h and remained high for all the analyzed times. On the other hand, PA treatment led to a subsequent decrease in the Δψm to baseline levels after 6 and 18 h, followed by a second rise in the Δψm, noticeable at 24 h, thus suggesting a two-phase response for this fatty acid.

Next, we assessed the O_2_ consumption rate (OCR) as a measure of mitochondrial oxidative activity. Treatments with OA or PA increased basal OCR but with different increment profiles (Figure 4D). In the case of OA, the increment was slower, becoming significant at 6 h. PA triggered a faster increase in the OCR, as early as 2 h, which continued at 6 h (Figure 4D). We also measured OCR in the presence of oligomycin, an inhibitor of ATP synthesis, which reveals the amount of mitochondrial respiration not associated with ATP production (Figure 4D). Neither OA nor PA changed the OCR in the presence of oligomycin, thereby suggesting that neither treatment affects the mitochondrial coupling between respiration and ATP production. In other words, it appears that mitochondria remain similarly “efficient”. Additionally, we also measured OCR in the presence of FCCP, which dissipates the proton gradient at the inner mitochondrial membrane (i.e., Δψm). Given that mitochondrial respiration is a process that works against the proton gradient, the elimination of the Δψm allows respiration to reach its maximum levels. As with oligomycin, neither OA nor PA changed the magnitude of FCCP-induced OCR (Figure 4D), implying that mitochondria maintain a similar “maximal performance” across treatments. Taken together, these results suggest that the changes in basal OCR would not be due to differences in mitochondrial functional capacity (for example, increases in mitochondrial functional units or damage of existing ones), but rather to regulatory changes, such as differential substrate availability or upstream signaling cascades.

Similar to OCR, PA treatment triggered a significant increase in ATP production as soon as 2 h, which remained high at least until 6 h. However, OA treatment led to a slower ATP increase, which reached significance at 6 h (Figure 4E). As an operational indicator of the mitochondrial functional profiles, we used the relationship between ATP production and the Δψm (Figure 4F). At 2 h, the ATP/Δψm ratio remained relatively constant for both conditions. Nonetheless, at 6 h there was a divergence: the mitochondrial network from PA-treated cells appeared more ATP production-oriented, while the network from OA-treated cells seemed to prefer Δψm build-up. Altogether, these results suggest that both treatments stimulate mitochondrial bioenergetics, but with different functional profiles.

### Oleic and Palmitic Acids Stimulate Divergent Specialization of Mitochondrial Populations

**Figure 5:**
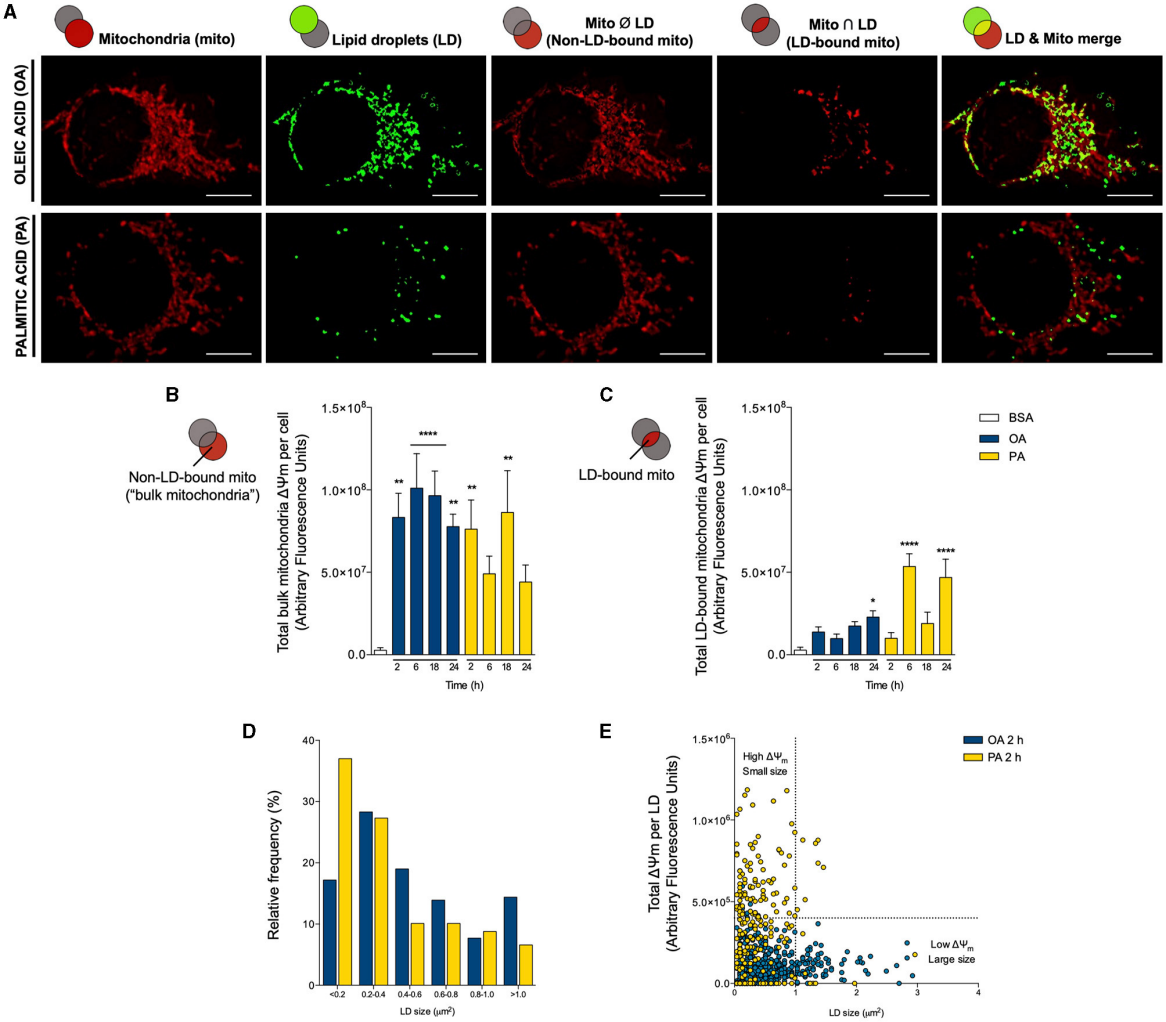
Oleic and palmitic acids evoke different patterns of lipid droplet-mitochondria physical and bioenergetic coupling in HepG2 cells. **(A)** Representative confocal images of HepG2 cells treated with 200 μM of oleic acid (OA) or palmitic acid (PA) during 6 h and stained with Bodipy 493/503 (green) and MitoTracker Orange (red) for lipid droplet (LD) and mitochondria visualization, respectively. Individual organelles are shown in the first two columns, from left to right. The third column shows the intersection between LD and the mitochondrial network; that is, LD-mitochondria interaction. The fourth column shows the mitochondrial network minus the LD system; that is, non-LD-bound mitochondria or “bulk mitochondria”. The fifth column displays the merge between the first two rows. **(B,C)** Total Δψm of “bulk mitochondria” and LD-bound mitochondria of HepG2 cells treated as in **A** for 0–24 h, measured using MitoTracker Orange and confocal fluorescence microscopy. Data are from *N* = 6–20 cells analyzed from three independent experiments. Scale bar: 10 μm. Results are shown as mean ± SEM; ^*^p < 0.05; ^**^p < 0.01; and ^****^p < 0.0001 vs. BSA. **(D)** Histogram of LD size distribution of cells treated as in **A** for 2 h. **(E)** Dot plot of the size of each LD vs. the Δψm of associated mitochondria in cells treated as in **A** for 2 h. The vertical dotted line cuts off small LD (left) from larger ones (right), while the horizontal line divides LDs between those associated with lower (bottom) and high (top)-Δψm mitochondria.

In sum, both PA and OA fatty acids induce distinct changes in mitochondrial morphology, LD-mitochondria interaction, and their bridging proteins and at the level of mitochondrial functionality. However, our previous studies have shown that the mitochondrial network and its interactions are highly heterogeneous (22, 23). Thus, the observed changes probably involve a subpopulation of mitochondria as a sort of “specialization mechanism”. Moreover, mounting evidence underscores an association between mitochondrial size and function, although in a cell type-dependent fashion (35). In brown adipocytes, which avidly oxidize FA, Benador et al. showed that LD-associated mitochondria are larger in size and mainly dedicated to ATP production and LD expansion, while smaller mitochondria associate mainly with FA oxidation (8). To address whether OA or PA replicate this behavior in our hepatocyte-derived cell line, we first compared the Δψm level of the LD-bound mitochondrial network with the rest of the mitochondrial network (hereafter termed “bulk mitochondria”). In Figures 5A,B, we show that although both OA and PA increase the Δψm of bulk mitochondria starting at 2 h, only OA has a steady effect. In contrast, PA again displays a biphasic response, which faded at 6 h, reappeared at 18 h, and finally disappeared after 24 h. In terms of LD-bound mitochondria, OA caused a minor accretion in their Δψm, which became significant after 24 h of treatment (Figure 5C). On the other hand, PA augmented the Δψm after 6 h, which faded at 18 h, but reappeared at 24 h. Curiously, the Δψm increments in bulk mitochondria interleaved with those of the LD-bound mitochondria. Taken together, these results suggest that OA induces steep nucleation and gradual expansion of LDs, which strongly interact with mitochondria exhibiting a relatively “weak” Δψm. Contrarily, PA triggers the biogenesis of multiple but small LDs, which show a moderately increased interaction with mitochondria harboring a relatively “strong” Δψm, associated with higher OCR and ATP production.

Finally, to assess the association between mitochondrial bioenergetics and LDs dynamics, we correlated the size of each individual LD with the Δψm of the associated mitochondria. We chose the temporality of 2 h because this is our earliest determination of LD nucleation, where the differences between OA and PA are most marked. In agreement with Figure 1, the histogram of Figure 5D shows that OA favors the emergence of larger LDs (cross-sectional area > 0.2 μm^2^), while PA leads to the accumulation of smaller ones with smaller areas (< 0.2 μm^2^). Then, we analyzed each LD from 18-20 cells in both conditions and correlated the size of each LD with the associated total MitoTracker Orange fluorescence (Figure 5E). That is, we evaluated how active were the mitochondria associated with each LD (in terms of the Δψm). We found that the different fatty acids promoted the appearance of distinct populations of LDs, respective to their size and the activity of the attached mitochondria. In either case, the “bulk LDs” consisted of units with smaller size (< 1 μm^2^), associated with mitochondria with lower Δψm. As initially noted, OA led to the emergence of larger LDs, characterized by their colocalization with mitochondria with lower Δψm. On the contrary, PA treatment did not promote the enlargement of LDs but triggered the appearance of a sub-population of small-sized LDs associated with mitochondria with higher Δψm. Altogether, these observations indicate that the unsaturated fatty acid OA promotes lipid accumulation in larger LDs proximal to mitochondria that build-up lower Δψm and that the saturated fatty acid PA favors smaller LDs in close apposition to mitochondria with higher Δψm.

## Discussion

**Figure 6:**
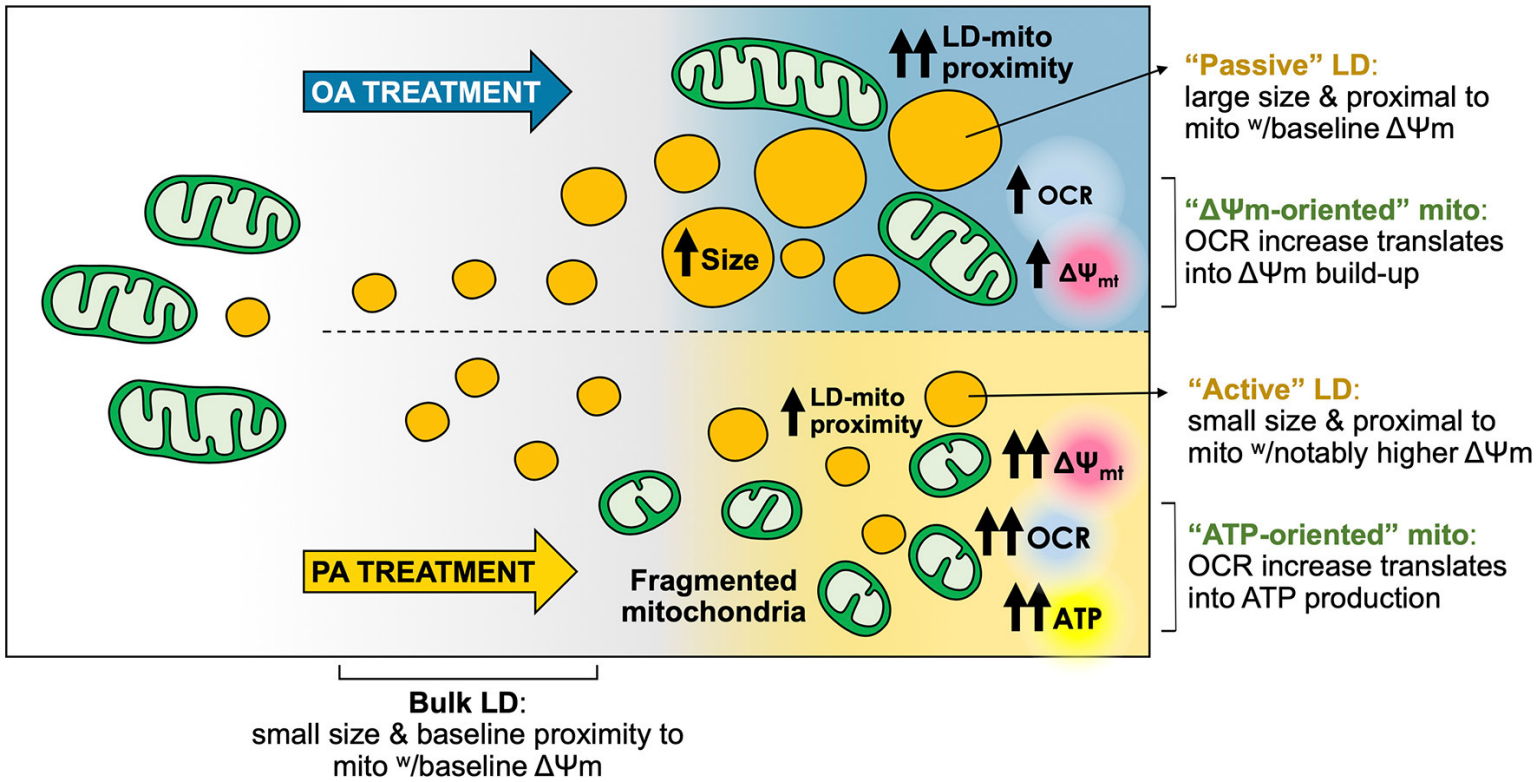
Oleic and palmitic acids favor different lipid droplet and mitochondria sub-populations with distinct morphological and bioenergetic profiles in HepG2 cells. Both oleic acid (OA) and palmitic acid (PA) induce the formation of lipid droplets (LDs) in HepG2 cells. OA induces a steep nucleation process, followed by a steady growth of LDs. PA stimulates a moderate increase in the number of LDs, which maintain a small size. Moreover, while OA does not affect mitochondrial morphology, PA elicits fragmentation of the mitochondrial network. Both treatments boost mitochondrial respiration (OCR), but with different outcomes. OA mainly heightens the overall transmembrane potential of the mitochondrial network (Δψm-oriented network). On the other hand, PA treatment favors ATP generation (ATP-oriented network). LD-mitochondria physical proximity increases upon treatment with either fatty acid. OA evokes higher levels of LD-mitochondria interaction compared to PA. However, LDs from OA-treated cells are rather “passive”, as their larger size associates with lower-Δψm mitochondria, compared to PA. LDs from PA-treated cells are apparently more “active”, as they are smaller and interact with higher-Δψm mitochondria, which is concomitant with increased ATP levels.

Within the cell, FA have structural and functional roles, and serve as an energy reservoir stored in the form of TG. The latter is essential to ensure a continuous FA supply, independent of external nutrient availability (36). However, high-fat diets associate with obesity and other related diseases (37), with several studies showing that the monounsaturated FA OA is less toxic than the saturated FA PA. Even more, OA can prevent PA-induced toxicity in hepatocytes (5–7), although the differential impact in LD-mitochondria dynamics and mitochondrial bioenergetics of each FA was not explored. Here, we report that PA and OA elicited a differential cellular response in the hepatocyte cell line HepG2. While both fatty acids led to massive TG accumulation, OA promoted the formation of more and bigger LDs, which were in closer contact with mitochondria compared to PA. Instead, the latter triggered a fast and intense process of mitochondrial fragmentation associated with increased OCR and ATP levels. Interestingly, the large LDs promoted by OA treatment were proximal to mitochondria with a baseline Δψm (“passive LDs”). In contrast, PA promoted the formation of small-sized LDs proximal to mitochondria with enhanced Δψm (“active LDs”) (Figure 6). This feature may contribute to explain the toxic effects of saturated FA, like PA, on hepatocytes.

The effect of OA promoting bigger and more abundant LDs compared to PA (Figure 1) (5) has also been observed in other cell types, such as pancreatic β-cells (38), chondrocytes (39), and H9C2 cardiomyoblasts (40). This greater capacity of OA to be esterified to TG and accumulated into LDs likely explains its reduced lipotoxicity compared to PA. Furthermore, OA reportedly ameliorates PA-induced toxicity in hepatocytes (5, 7, 41), although it is unclear whether OA promotion of PA redirection to TG is the primary protective mechanism (6). For instance, OA also induces the redistribution of ceramide synthases to LDs. These enzymes catalyze the storage of ceramide as acylceramide, thereby decreasing the toxic effect of ceramide accumulation (42).

LDs participate in controlling energy metabolism and thus communicate with other organelles, relying on regions of close contact, including mitochondria, ER, and peroxisomes (9). The LD-mitochondria association has been reported particularly in tissues with a high TG oxidation and storage capacity, such as liver, heart, brown adipose tissue, and skeletal muscle (8, 13, 30). Notably, starvation-induced LDs in cardiomyocytes require a fused mitochondrial network to ensure mitochondrial oxidation of FA (43), suggesting that LD and mitochondrial dynamics and function are highly interrelated. Our study, like others, shows that PA triggers rapid mitochondrial fragmentation. On the other hand, OA treatment induced a slower increase in mitochondrial fragmentation, which was not as marked as the one induced by PA (Figure 2). These results support the idea that saturated and unsaturated FA differentially affect the mitochondrial fusion/fission equilibrium.

LD-mitochondria physical contacts are observable from yeast to mammalian cells (9), and their extent varies according to lipolytic stimuli (17, 44), exercise (45) or starvation (43, 46). Under starvation conditions, LD-mitochondria contacts increase, which supports FA transfer to mitochondria for β-oxidation (43). Similar results were observed in brown adipocytes, where cold exposure increased LD-mitochondria interaction, supporting thermogenesis (14, 40). More recently, Benador et al. reported in brown adipocytes that mitochondria in close contact with LDs maintain their oxidative capacity but have low levels of FA oxidation, thereby supporting LD growth by providing ATP for TG synthesis (8). Hence, two models of LD-mitochondria coupling have been so far described: the interaction that favors LD consumption and energy production, and the interaction that prompts LD expansion (30, 47). In the hepatocyte *in vitro* model presented here, we found that OA promotes a steeper increase in LD-mitochondria contacts than PA (Figure 2), suggesting that in HepG2 cells, OA feeding evokes the type of LD-mitochondria that mediates LD expansion.

Remarkably, only OA treatment increased PLIN5 and MNF2 protein levels, which putatively support the observed increment in LD-mitochondria contacts (Figure 3). Reportedly, PLIN5 is present in multiple cells and tissues, including hepatic and heart muscle cells (48). In accordance with our results, hepatocytes from liver-specific PLIN5 KO mice display fewer LD-mitochondria contacts, reduced hepatic TG synthesis and FA oxidation, and are more susceptible to develop hepatic insulin resistance (49). On the contrary, mice overexpressing liver-specific PLIN5 fed with a high-fat diet exhibit severe steatosis without worsening glucose homeostasis. Surprisingly, these animals have lower fasting insulin levels, suggesting preservation of insulin sensitivity (50). Similar to PLIN5, MFN2 is needed for LD-mitochondria interaction, as shown in brown adipose tissue, where it supports FA-fueled thermogenesis (51). Accordingly, MFN2 ablation protects against high-fat diet-induced insulin resistance in brown adipose tissue (17). On the other hand, in our study model, PA treatment only raised PLIN2, but not PLIN5 nor MFN2 protein levels. This observation agrees with another study showing that PLIN2 promotes liver steatosis in mice (52). However, how PLIN2 participates in LD-mitochondria coupling remains to be unveiled. Altogether, our observations indicate that OA and PA treatments induce different sets of LD-mitochondria tethers, which underlie the distinctive functional coupling between both organelles.

Additionally, our study showed that PA treatment triggers a faster and more pronounced mitochondrial fragmentation than OA, concomitant with markedly higher OCR, Δψm and ATP levels (Figures 2, 4). These results support the idea that saturated and unsaturated fatty acids differentially regulate the mitochondrial fusion/fission equilibrium and bioenergetics, generating two mitochondrial networks with distinct metabolic profiles. This agrees with the study of Benador et al. in brown adipocytes, in which smaller mitochondria contributed to fatty acid oxidation more actively, while larger mitochondria mainly participated in LD growth by providing ATP for the synthesis of TG (8). These divergent mitochondrial behaviors explain not only the differences in LD morphology after PA and OA treatments but also different metabolic parameters (Figure 4F): PA oxidation greatly fuels OCR and ATP production (i.e., ATP-oriented mitochondrial network), while OA accumulation induces a limited increase in OCR and ATP, leading to Δψm accretion (i.e., Δψm-oriented mitochondria network). These dissimilar metabolic fates partially explain the higher toxicity of saturated FA in hepatocytes compared to unsaturated FA.

Consistent with our aforementioned interpretation, the analysis of LD-mitochondria proximity showed that OA-induced LD is more extensively proximal to mitochondria than PA (Figure 5). However, these mitochondria maintain a baseline metabolic activity, as indicated by MitoTracker Orange staining. Thus, we hypothesized that OA-induced LD does not fuel oxidative metabolism, but instead consumes ATP, thus acting as “passive” LDs in terms of mitochondrial bioenergetics. Interestingly, LD-bound mitochondria under OA treatment displayed a lower Δψm compared to the rest of the mitochondrial network. This can be explained by the fact that the ATP consumption required for LD growth contributes to the dissipation of the Δψm. On the other hand, PA promoted the accumulation of small-sized LDs proximal to mitochondria with higher Δψm (Figure 5). Accordingly, we speculate that these LDs fuel mitochondrial bioenergetics, thus acting as metabolically “active” LDs that do not particularly consume ATP, thus leading to Δψm accumulation in the mitochondria that stand nearer.

Reportedly, mitochondria associate with LDs in tissues exhibiting a high capacity for TG storage and oxidation, such as the liver, heart, brown adipose tissue, and skeletal muscle (8, 13, 30). Notably, contrasting evidence has shown that mitochondrial oxidation of LD-derived fatty acids requires mitochondria fusion in MEF cells (43) or fission in brown adipocytes (8), suggesting that LD metabolism and mitochondrial dynamics are interrelated, and seemingly dependent on the cell type. Moreover, our study also showed that the fatty acid type also determines the LD/mitochondrial dynamics ratio.

## Conclusion

In sum, our data uncover two patterns of LD-mitochondria interaction in response to treatment with two different fatty acids. On the one hand, OA led to triglyceride accumulation, concomitant with increased LD-mitochondria proximity. Under this condition, the overall mitochondrial network underwent only a slight metabolic boost, which mainly exerted a Δψm-oriented role instead of contributing to ATP production. Meanwhile, OA-induced LD appeared rather “passive”, precluding fatty acids from mitochondrial oxidation, thereby thwarting the bioenergetic boost of nearby mitochondria. On the other hand, PA-induced a slight increase in LD accumulation. Newly formed LDs were seemingly “active” and associated with mitochondria with higher OCR and Δψm, compared to the rest of the network (Figure 6). Thus, we hypothesized that PA treatment renders mitochondria more ATP-oriented compared to OA treatment, due to higher substrate availability. Our results underscore the importance of the dietary FA composition in the development of NAFLD, where saturated FA promote hepatic steatosis with mitochondrial dysfunction, which in turn can promote NAFLD progression.

## Data Availability Statement

The raw data supporting the conclusions of this article will be made available by the authors, without undue reservation.

## Author Contributions

RT and VP conceived and designed the study. AE, FD-C, and JB performed the experiments. AE, RB-S, and VP analyzed the data. AE, VP, and RT interpreted the data. AE, RB-S, VP, and RT drafted the manuscript reviewed by all authors. All authors contributed to the article and approved the submitted version.

## Funding

This work was supported by grants from the Agencia Nacional de Investigación y Desarrollo (ANID), Chile: FONDECYT 1191078 to RT; 1190743 to VP; 11201267 to RB-S; FONDAP 15130011 to RT, VP, and RB-S; PAI Insertion Program 77170004 to RB-S; University of Chile U-inicia UI-006/19 to RB-S and U-redes 2018-G_2018-35 to VP; and CRP-ICGEB CHL18-04 to VP.

## Conflict of Interest

The authors declare that the research was conducted in the absence of any commercial or financial relationships that could be construed as a potential conflict of interest.

## Publisher's Note

All claims expressed in this article are solely those of the authors and do not necessarily represent those of their affiliated organizations, or those of the publisher, the editors and the reviewers. Any product that may be evaluated in this article, or claim that may be made by its manufacturer, is not guaranteed or endorsed by the publisher.

## Abbreviations

ATP: Adenosine triphosphate
BSA: Fetal bovine serum
ER: Endoplasmic reticulum
ETC: Electron transport chain
FA: Fatty acids
FCCP: Carbonyl cyanide 4-(trifluoromethoxy)phenylhydrazone
GAPDH: Glyceraldehyde-3-phosphate dehydrogenase
LD: Lipid droplets
MFN2: Mitofusin 2
MIGA2: Mitoguardin 2
NAFLD: Non-alcoholic fatty liver disease
OA: Oleic acid
OCR: Oxygen consumption rate
OPA1: OPA1 mitochondrial dynamin-like GTPase
PA: Palmitic acid
PBS: Phosphate-buffered saline
PLIN: Perilipin family of proteins
PLIN1: Perilipin 1
PLIN2: Perilipin 2
PLIN5: Perilipin 5
TG: Triglycerides
Δψm: Mitochondrial transmembrane potential.
